# Chronic Urticaria: The Need for Improved Definition

**DOI:** 10.3389/falgy.2022.905677

**Published:** 2022-06-09

**Authors:** R. Maximiliano Gómez, Jonathan A. Bernstein, Ignacio Ansotegui, Marcus Maurer

**Affiliations:** ^1^Fundación Ayre, UCARE Center, Salta, Argentina; ^2^School of Health Sciences, Catholic University of Salta, Salta, Argentina; ^3^Internal Medicine Department, University of Cincinnati, Cincinnati, OH, United States; ^4^Allergy and Immunology Department, Hospital Quironsalud Bizkaia, Bilbao, Spain; ^5^Allergy Centre Charité, University of Berlin, Berlin, Germany

**Keywords:** chronic urticaria, diagnosis, opinion, revision, dissemination, epidemiology

## Looking for Diagnostic Certainty on Urticaria

The diagnosis of CU requires the presence of daily or almost daily presence of urticaria persisting for 6 weeks or longer, irrespective of whether it is spontaneous (CSU) or inducible (CIndU) in nature, compared to acute urticaria that lasts <6 weeks. Urticaria is identified by the presence of pruritic wheals or hives, accompanied by angioedema (AE) in ~40% of cases. Up to 20% of patients present with isolated AE (1–3).

The definition of wheals or hives involves the presence of polymorphic raised skin lesions that are rounded or irregular in shape, with a pale central region and erythematous borders (although complete redness of wheals may occur), which usually persists for several hours but less than a day. These later two characteristics can sometimes help differentiate CU from vasculitis lesions, as they typically persist over 24 h and usually have a hematoma appearance (4). In addition, an uncomplicated urticaria lesion will disappear by applying pressure to the lesions whereas urticaria vasculitis lesions typically persist (1, 3, 5).

The typical histopathologic features of urticarial wheals exhibit lymphocytic infiltrates with perivascular eosinophils however, there may be mixed infiltrates of eosinophils and neutrophils, which is more often associated with chronic autoimmune urticaria.

Mast cells, which require special staining to visualize (i.e., Tryptase, CD117) are increased up to 10-fold the number found in normal skin in the reticular dermis. The presence of not only extravasated erythrocytes but also fibrinoid necrosis and leukocytoclasia are suggestive of leucocytoclastic vasculitis, which may be associated with normal or decreased complement levels (6, 7).

The definition of AE corresponds to vascular permeability in the deeper subcutis region that is typically non-pruritic but sometimes painful due to increased fluid accumulation in the interstitial space causing increased pressure and innervation of nerve fibers. Histaminergic AE which may or may not be associated with urticaria (isolated histaminergic AE), typically resolves within 72 h without treatment. Non-histaminergic AE is often bradykinin-mediated and not associated with urticaria, may persist longer than 72 h and in severe cases progress to asphyxia of the upper airway without treatment. Bradikinin-mediated AE is associated with ACE-inhibitors or different forms of hereditary or acquired AE. Histaminergic AE in contrast to non-histaminergic forms is typically not associated with a family history of AE, with gastrointestinal symptoms and is responsive to treatment with corticosteroids and antihistamines (8, 9).

Table 1 summarizes clinical characteristics of urticaria that can help clinicians properly diagnose this condition.

**Table 1:** Clinical characteristics of urticaria.

	**Presence**	**Comments**
Wheals/Hives	+++	Heterogeneous in size and shapes; can be papular or macular
Duration of wheals	+	Limited to hours—evanescent
Lesions disappear when pressure applied	+++	Contrasts to vasculitis
Itching/Pruritus	++	Often intense
Angioedema	++	Can be isolated
Duration of angioedema	+	Resolves without treatment within 72 h

## Is CU a Matter of Concern?

The significant burden of CU has been extensively reported with a variety of validated patient reported outcome measures that demonstrate a significant impact on several aspects of life ranging from physical discomfort to personal mood changes (particularly anxiety and depression) which often interferes with interpersonal relationships, daily activities including work and school. Not surprisingly, management of CU is associated with substantial costs to our health care system due to frequent medical visits and therapies (4, 10, 11). Therefore, it is imperative to create awareness among healthcare payors and other stakeholders about the prevalence of CU and its impact on patient quality of life and the economic burden it has on society. The lack of consensus on diagnostic criteria for CU makes this task more challenging to achieve. Nonetheless, advances in the medical recognition of allergic and immunological conditions such as CU by the International Classification of Diseases (ICD)-11 committee can help overcome such barriers to ensure this condition is correctly identified by medical practitioners (12, 13).

Currently, the prevalence of CU is estimated to range from <1% to over 5% in general population (4, 11, 14) indicating that hundreds of millions of people are affected by this condition. Moreover, during the recent COVID pandemic many more individuals experienced cases of acute urticaria and there was a remarkable surge of urticarial exacerbations among patients with existing CU (15).

Registries are important for identifying existing and new cases of CU [(16), https://www.urticaria-registry.com/registry.shtml] and currently there is an ongoing voluntary, observational open registry for CU that allows any physician to provide baseline and follow-up data on their CU patient's demographics, symptom characterization, triggers, associated risk factors, comorbid conditions and treatment (17). In addition, a cross sectional registry from Latin America has been useful in obtaining retrospective data on real-life management and outcomes of CU patients (18). A multicentric study comparing CU patients from Europe and Latin America found that CU patients from Europe were less likely to present with angioedema or experience concomitant chronic spontaneous urticaria with an inducible component (CIndU). In addition, they had higher rates of controlled disease and better overall treatment access but interestingly they still had significantly impaired quality of life parameters (19). Data from registries can be used to confirm such findings.

Chronic urticaria is a heterogeneous condition and its duration varies between individuals which makes it more challenging to estimate its prevalence. A prospective evaluation of CU patients found that around 50% of those with CSU experienced remission within 1 year after onset, compared to <20% of those with CIndU. In general, patients with more severe disease and inducible triggers (CIndU) took much longer to achieve complete remission (20). A more recent report describes that up to 80% of patients with CSU may achieve remission over 1 year but >10% may suffer a more prolonged time course of up to 5 years (3). This variability in disease remission can be explained by several factors and comorbidities that have led to the categorization of patients into specific clinical phenotypes based on the presence or absence of certain inflammatory cells or autoantibodies predicting a good or poor response to high dose antihistamines or biologics. These phenotypes have subsequently been linked to endotypes that have improved our understanding of the underlying immunopathomechanisms of this complex condition and have led to the development of more targeted therapies that could potentially improve the management of CU patients refractory to current treatment options. Current CU guidelines represent living documents that will continuously be modified as our knowledge about CU continues to broaden (5, 21).

Limitations were exposed along the present document, about the absence of a consensus on diagnostic criteria, while its strength is the proposal of practical parameters for an undoubtful identification of CU.

## Conclusions

The health and economic burden of CU is substantial and should not be trivialized. The significant impact of CSU on patients requires that physicians and other health care providers understand how to properly identify and manage this condition. An expert consensus on diagnostic criteria for CU is urgently required to improve the reliability of global epidemiologic data.

## Author Contributions

All authors listed have made a substantial, direct, and intellectual contribution to the work and approved it for publication.

## Funding

Ayre Foundation benefactor covered publication costs.

## Conflict of Interest

The authors declare that the research was conducted in the absence of any commercial or financial relationships that could be construed as a potential conflict of interest.

## Publisher's Note

All claims expressed in this article are solely those of the authors and do not necessarily represent those of their affiliated organizations, or those of the publisher, the editors and the reviewers. Any product that may be evaluated in this article, or claim that may be made by its manufacturer, is not guaranteed or endorsed by the publisher.
